# Cardiac Aneurysms

**Published:** 1903-10-17

**Authors:** 


### Hospital Clinics and Medical Progress.

### CARDIAC ANEURYSMS.

Dr. D. G. Hall[1] contributes a valuable paper on this subject which, as he says, "must necessarily be of interest rather to the pathologist than to the clinician, for they (cardiac aneurysms) are outside the pale of practical diagnosis, and beyond the reach of treatment." He makes the following classifications: (1) Aneurysms of the cardiac walls; (2) aneurysms of the cardiac valves; (3) aneurysms of the cardiac (coronary) arteries. Dr. Hall has collected 112 cases of aneurysm of the cardiac walls, in which the site of the aneurysm was as follows: left ventricle, 92 cases; right ventricle, one case; left auricle, two cases; ventricular septum—(*a*) muscular part, eight cases; (*b*) membranous part, seven cases; auricular septum, two cases. Aneurysm of the left ventricle is thus seen to be far more common than that of the other three chambers of the heart combined, and may be taken as the type of aneurysm of the cardiac wall. The commonest site is the apex of the ventricle or the anterior wall just above the apex. The condition is more frequently met with in males than females, the proportion being about three to one. There is seldom more than one aneurysm present. The aneurysm is not usually visible until the heart is cut open, when it appears as a depression hollowed out at the expense of the thickness of the heart wall. The depression in some instances is not sharply defined, in others there is a marked constriction at the neck where the aneurysm opens into the cavity of the ventricle. In the latter form there is nearly always laminated clot to be found. Pericardial lesions are nearly always present, most commonly in the form of adhesion between the two layers in the region of the aneurysm, though sometimes there is merely increased vascularity and thickening of the visceral layer. The endocardium of the aneurysm is invariably altered, as shown by thickening and loss of lustre and transparency. The myocardium is essentially affected, the change being either of the nature of an acute lesion—such as partial rupture, hæmorrhage, or infarction—or fatty degeneration, which may be considered as an acute lesion produced by interference with the blood supply, or a chronic lesion such as fibrosis, which has long been recognised as the great cause of these aneurysms. In old standing cases the fibrous tissue may become the seat of calcification. Whether, as has been urged, fibrous myocarditis is the invariable precursor of aneurysm of the heart, or whether, as some pathologists believe, fatty degeneration may sometimes be the cause, does not much matter, for, as Dr. Hall points out, both these forms of degeneration have a common origin in obstruction of the coronary arteries. The view has been advanced that the primary weakening of the muscular wall is due to a myocarditis spreading inwards from the pericardium, and that the pericardial adhesion is a forerunner, instead of the result of aneurysm. Dr. Hall believes it to be much more likely that the pericardial adhesion is a result of the aneurysm, and this view is supported by the fact that in some cases of aneurysm there is no pericardial adhesion at all, while the pericardial theory does not altogether explain the frequency of origin of the aneurysms at the apex of the left ventricle.

It is to coronary obstruction that Dr. Hall attributes the chief part in the causation of cardiac aneurysm. In 51 of his collected cases the condition of the coronary arteries is mentioned. In 5 of these the arteries are reported as healthy; in 17 there was atheroma without any mention of complete obliteration; in 29 one or both coronary arteries are reported as being completely blocked. In every case where obstruction had occurred there was pre-existing disease of the arterial walls. The frequency with which the apex of the heart is the site of the aneurysm is accounted for by the fact that it is the most distal part, so that obstruction of the vessel at whatever level is sure to affect the apex. Then, again, the apex is the thinnest part of the left ventricle, the chamber of the heart in which the blood pressure is the highest. Though coronary obstruction is the commonest cause of these aneurysms there are others, *e g*, trauma, myocarditis due to extension of inflammation from a diseased valve, gumma, cysts in the heart wall.

The diagnosis is not likely to be made during life. In only one of his collected cases was it even suggested before death that the patient might have a cardiac aneurysm. The sign which has been most commonly present is a gallop rhythm due to reduplication of the second heart sound; attacks of angina pectoris are not uncommon. Death may occur from rupture of the aneurysm, angina pectoris, pulmonary œdema, or gradual heart failure.

Aneurysms of the cardiac septa usually occur in the part of the interventricular septum which is normally fibroid and which is situated in the highest part of the septum between the posterior and right semilunar valves. This congenitally weak spot is known as the "undefended space." This space is crossed by the tricuspid valve, and as aneurysms here practically always project into the right heart, they may protrude above the tricuspid valve into the right auricle, below the tricuspid valve into the right ventricle, or pushing forward the valve may bulge into both cavities. The aneurysm may give rise to no symptoms at all, or there may be evidence of some cardiac lesion, dyspnœa being fairly common. Aneurysms of the cardiac valves are almost limited to the left side of the heart, and are slightly more common in the aortic than in the mitral valve. In very few of the reported cases is any satisfactory history given. Acute rheumatism is a factor in the causation of some instances. Aneurysms of the cardiac valves are rarely of large size as they usually rupture early. In the event of congenital fusion of two aortic cusps the malformed segment is particularly liable to disease. The large anterior cusp of the mitral valve is moie commonly affected than the other two. In a total of 41 cases of mitral aneurysms the large cusp was affected in 36. Although the frequency of atheroma of the coronary arteries is remarkable, yet it is very rarely that they are afflicted by aneurysm. It appears that aneurysm of these vessels, as in the case of other small arteries, is due to septic embolism. The position of the orifices of the coronary arteries explains the infrequency of embolism in these vessels. The heart itself is nearly always the source of these emboli, which are due to infective vegetative endocarditis. Another reason of the rarity of aneurysm of the coronary arteries is that an embolus of sufficient size to cause an aneurysm is likely to cause sudden death. These aneurysms show a marked tendency towards rupture. In 11 out of the 25 cases collected by Dr. Hall death was caused by rupture into the pericardium. Unless rupture occurs the aneurysm gives rise to no clinical manifestations.

[1] Edinburgh Medical Journal, October, 1903.

